# Efficacy and Safety of Endoscopic Esophageal Dilatation in Pediatric Patients with Esophageal Strictures

**DOI:** 10.1155/2021/1277530

**Published:** 2021-09-24

**Authors:** Hasan M. A. Isa, Khadija A. Hasan, Husain Y. Ahmed, Afaf M. Mohamed

**Affiliations:** ^1^Pediatric Department, Salmaniya Medical Complex, Arabian Gulf University, Manama, Bahrain; ^2^Pediatric Department, Salmaniya Medical Complex, Manama, Bahrain; ^3^Public Health Department, Ministry of Health, Manama, Bahrain

## Abstract

**Materials and Methods:**

In this retrospective cross-sectional single center study, records of patients with esophageal strictures presented to the pediatric department, Salmaniya Medical Complex, Bahrain, in the period between 1995 and 2019 were reviewed. Demographic data, indications of endoscopic dilatations, the procedure success rate, and possible complications were assessed.

**Results:**

Forty-six children were found to have esophageal strictures. Twenty-five (54.3%) patients were males. Most patients presented during infancy (86.5%, 32/37 patients). Twenty-six (56.5%) patients required 88 dilatation sessions, while the remaining 20 (43.5%) patients did not require dilatations. The median number of dilatation sessions per patient was three (interquartile range = 2–5). Savary-Gilliard bougienages were the main dilators used (80.8%, 21/26 patients). Anastomotic stricture (post esophageal atresia/tracheoesophageal fistula repair) was the main cause of esophageal strictures and was found in 35 (76.1%) patients. Patients with nonanastomotic strictures had more frequent dilatations compared to those with anastomotic strictures (*P* = 0.007). The procedure success rate was 98.8%. Yet, it was operator dependent (*P* = 0.047). Complete response to dilatation was found in 18 (69.2%) patients, satisfactory in seven (26.9%), and an inadequate response in one (3.9%). Those with satisfactory responses still require ongoing dilatations based on their symptoms and radiological and endoscopic findings. No perforation or mortality was reported. Patients with dilatations had more recurrent hospitalization (*P* < 0.0001), more dysphagia (*P* = 0.001), but shorter hospital stay (*P* = 0.046) compared to those without dilatations. Surgical intervention was required in one patient with caustic strictures. The median follow-up period was six years (interquartile range = 2.25–9.0).

**Conclusions:**

Endoscopic esophageal dilatation in children with esophageal strictures is effective and safe. Yet, it was operator dependent. Nonanastomotic strictures require more dilatations compared to anastomotic strictures. Findings of this study are comparable to those reported worldwide.

## 1. Introduction

Benign esophageal stricture is the main cause of esophageal stricture in children. Management of these strictures includes medical therapy, esophageal dilatation, and surgical intervention [1].

Esophageal dilatation is the mainstay therapy for benign strictures [2]. It can be performed either endoscopically or fluoroscopically [3]. The endoscopic dilatation is the most frequent approach used in children and adults [1, 2, 4–8]. Fluoroscopic-guided dilatation is another treatment option but it carries the risk of radiation exposure [1].

The most possible mechanisms of the dilatation are either splitting of the stricture or circumferential stretching [7]. These are performed to interfere with the remodeling of the scar before it became stiff [7]. Currently, three types of esophageal dilators are in practice: wire-guided Savary-Gilliard bougienages (SGB), weighted Maloney dilators, and endoscopic balloon dilators (EBDs) [1, 6, 7, 9]. Endoscopic dilatations using SGB is the traditional therapy of esophageal strictures in children [10]. Yet, EBDs are increasingly recognized as a better option [1].

The success of the dilatation can be evaluated either directly through endoscopy (appearance of mucosal tearing at the stricture area) or indirectly through the fluoroscopic study (disappearance of the stricture's waist) [1].

Achieving sufficient food intake is the target of esophageal dilatation in patients with esophageal stricture [8]. If left untreated, patients with esophageal stricture may suffer from failure to thrive and can develop serious complications [3, 11]. The ingested food bolus may stick at the proximal part of the stenosis, compressing the membranous portion of the trachea leading to apnea and hypoxia [11]. Moreover, recurrent aspirational pneumonia can be fatal [12]. Endoscopic dilatation is an effective procedure to guarantee normal growth [8, 10, 13]. However, it might lead by itself to severe complications [14].

Upon reviewing the literature, there were only three studies about esophageal dilatations from the Arabian Gulf region, two from Saudi Arabia and one from Iran [1, 15, 16]. No studies about endoscopic dilatation of esophageal strictures in children came from Bahrain. This study is aimed at reviewing the efficacy and safety of endoscopic esophageal dilatations in pediatric patients presenting with esophageal strictures.

## 2. Material and Methods

### 2.1. Study Participants

In this retrospective cross-sectional single center study, a review of medical records for all patients diagnosed with esophageal strictures presented to the pediatric department, Salmaniya Medical Complex (SMC), Bahrain, in the period between August 1995 and August 2019 was conducted. SMC is the only tertiary center dealing with such patients in Bahrain. Patients older than 18 years at presentation were excluded. Patients were diagnosed to have stricture based on clinical, radiological, and endoscopic findings. The gastrografin study was used to detect esophageal strictures radiologically. Both Olympus and Pentax child gastroscopies were used. Esophageal dilatations were carried out using Savary dilators, balloon dilators, or both. Gastrointestinal endoscopy and esophageal dilatations were performed by two senior gastroenterologists. The first gastroenterologist was in service between 1995 and 2010, while the second gastroenterologist was working from 2011 to the time of this study. The choice of the dilatation technique depends on the cause of the stricture [5]. For example, inflammatory strictures secondary to gastroesophageal reflux or epidermolysis bullosa are better to be dilated with EBD, while SGB is more effective in patients with old narrowing strictures due to congenital stenosis [5]. Moreover, the selection of dilatation techniques can be affected by the severity of the stricture. Severe strictures can be dilated only with wire-guided SGB as the EBD cannot be passed through a much-narrowed lumen.

### 2.2. Data Collection

Demographic data including gender, nationality, gestational age, type of delivery, birth weight, age at presentation, age at the time of study, causes of esophageal stricture, level of stricture, and associated anomalies were collected.

Patients were divided into two groups according to the need for endoscopic esophageal dilatation. Patients who underwent endoscopic esophageal dilation were included in group 1. The decision of performing esophageal dilatation was taken if the standard or narrow diameter gastroscope (a diameter of 9 mm or 7 mm, respectively) did not pass through the narrowed esophageal lumen. The remaining patients who had symptoms that resolved with medical therapy alone without the need for endoscopy from the beginning or those who had persistent symptoms and underwent endoscopic procedure, but the scope passed through the narrowed segment without any resistance, so dilatation was not required, were included in group 2.

For the first group, the number of esophageal dilatations, year of the procedure, indication, and instrument used were identified. Different sizes of esophageal dilators were used. The wire-guided SGBs are polyvinyl tubes with sizes ranging from a 5 to 20 mm diameter. Balloon dilators have sizes ranging from 6 to 40 mm. In our center, SGBs of 5 to 11 mm diameter and balloon dilators of 8 to 14 mm diameter are available to be used in children, while larger sizes are reserved for adult patients. The gastroenterologist starts the dilatation procedure using the smallest dilator size and then gradually increases the size until successful dilatation was achieved. For SGB, a size of 5 mm is used initially followed by sizes of 7, 9, and 11 mm. For EBD, a size of 8 to 10 mm is the smallest size available to be used initially followed by 10 to 12 and 12 to 14 mm. Each patient will have one successful dilatation per session. The initial procedural success was attained by the appearance of esophageal mucosal tear.

Procedure efficacy and safety were assessed. The primary efficacy or success rate was defined as immediate postdilatation esophageal patency [17]. Complete response was defined as complete resolution of symptoms with no further dilatations required; satisfactory response was defined as a partial improvement of symptoms while an inadequate response was defined as no improvement of symptoms despite repeated dilatation session [1]. The use of proton pump inhibitors (PPIs) was documented.

The safety of the procedure was defined as a lack of complications during the first two days postdilatation [17]. Both groups were compared in terms of demographic data and overall complications.

The overall complications such as recurrent hospitalization (>one time), dysphagia, gastroesophageal reflux disease (GERD), failure to thrive, aspiration pneumonia, hospitalization (more than two weeks), apnea or dying spells, perforations, surgical interventions, postdilatation strictures, and the need for ongoing dilatations were retrieved. The nutritional status (thinness and stunting) was assessed based on the last visit patients' weight, height, and body mass index (BMI). Growth parameters were documented as a standard deviation (SD) from age- and sex-specific reference means. World Health Organization (WHO) growth standards for children from 2 to 5 years of age, school-age children, and adolescents from 5 to 19 years of age were used as references [18, 19]. Accordingly, thinness was defined as BMI < −2 SD while stunting was defined as height for age < −2 SD. Patients' survival and follow-up period were documented.

Outcomes of endoscopic esophageal dilatations (number of dilatations required, success rate, and complication rate) were assessed in relation to the type and location of esophageal stricture, dilatation technique, and the gastroenterologist who performed the procedures.

### 2.3. Statistical Analysis

The Statistical Package for Social Sciences (SPSS) version 21 program (SPSS Inc., Chicago, Illinois, USA) was used for analysis. Frequencies and percentages were calculated for categorical variables. Patients' ages were divided into the five year groups. Continuous variables were presented as mean and SD or median and interquartile range (IQR). Pearson chi-square and Fisher's exact tests were used to compare categorical variables. Student *t*-tests or the Mann–Whitney *U*-test was used to compare patients with or without dilatation in regard to birth weight and age at presentation. The Kruskal–Wallis test was used to compare frequency of esophageal dilatations in the last two decades (2000–2019) and to compare the number of dilatations needed by different sites of strictures and the dilatation techniques.

### 2.4. Ethical Approval

This study was in accordance with the principles of Helsinki Declaration, and it was ethically approved by the secondary care medical research subcommittee, SMC, Ministry of Health, Bahrain (IRB number: 75120521).

## 3. Results

**Figure 1:**
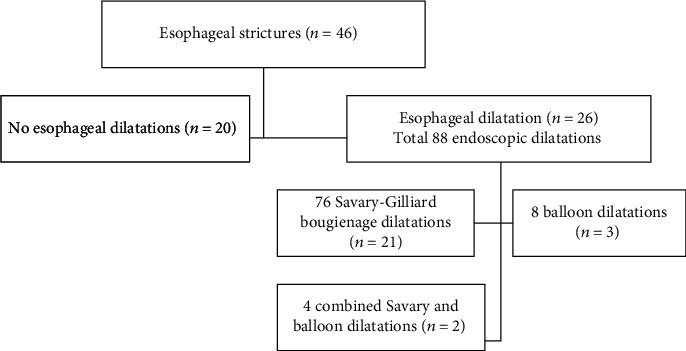
Endoscopic esophageal dilatations performed in children with esophageal strictures.

During the study period, 46 children were found to have esophageal strictures. Twenty-five (54.3%) patients were males and 21 (45.7%) were females. Most patients presented during infancy (86.5%, 32/37 patients). All the patients were symptomatic and had an evidence of esophageal stricture in the gastrografin study. Twenty-six (56.5%) patients required esophageal dilatations with a total of 88 dilatation sessions. The remaining 20 (43.5%) patients did not require dilatations (14 patients responded to medical therapy alone while eight patients underwent endoscopic procedure, but the scope passed without any resistance). The SGB dilator was used in 21 (80.8%) patients (Figure 1). The maximum dilator size used was 11 mm for SGD and 14 mm for EBD.

Demographic data of patients with or without esophageal dilatations are shown in Table 1.

Anastomotic post esophageal atresia/tracheoesophageal (EA/TEF) repair stricture was the main cause of esophageal strictures and was found in 35 (76.1%) of the patients. Both groups were comparable in all demographic data except for the etiology of esophageal strictures. The cause of esophageal stricture was anastomotic stricture in 16 (61.5%) patients requiring dilatation and 19 (95%) patients who did not require dilatation (*P* = 0.013). On the other hand, other causes such as gastroesophageal reflux, postcorrosive ingestion, and epidermolysis bullosa were found only in patients who required dilatations.

Out of the 46 patients, 35 (76%) patients had available data about the site of strictures, 26 (74.3%) had upper strictures, eight (22.9%) patients had middle strictures, and one (2.8%) had distal stricture.

**Figure 2:**
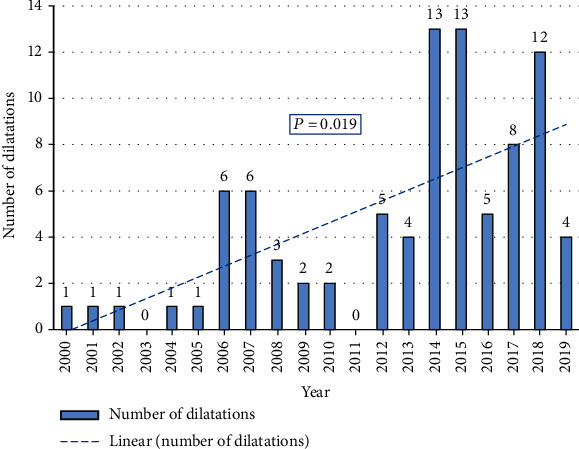
Numbers of endoscopic esophageal dilatations per year performed in children with esophageal strictures, 2000–2019.

Numbers of esophageal dilatations per year are shown in Figure 2.

The number of dilatations has increased in the last decade as the number of patients increased. The number of dilatations was four in 2000–2004, 18 in 2005–2009, 24 in 2010–2014, and 42 in 2015–2019. This rise was statistically significant (*P* = 0.019).

**Figure 3:**
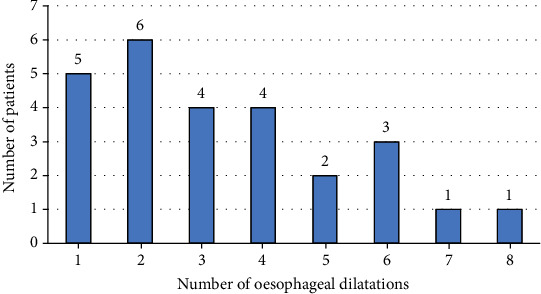
Numbers of endoscopic esophageal dilatations per patient performed for children with esophageal strictures.

Numbers of dilatation sessions per patient are shown in Figure 3.

The median number of dilatations per patient was three (IQR = 2–5), ranging from one to eight dilatations per patient. Patients with nonanastomotic strictures had more frequent dilatations (10 patients required 39 dilatations, mean ± SD = 4 ± 3) compared to patients with anastomotic strictures (16 patients required 49 dilatations, mean ± SD = 1 ± 2) (*P* = 0.007). There was no significant difference found between the different levels of strictures in terms of the number of dilatations required (*P* = 0.856).

The primary efficacy of the procedure was 98.8% (87 out of 88 dilatations). Complete response was found in 18 (69.2%) patients, satisfactory response in seven (26.9%), and an inadequate response in one (3.9%) patient. The latter was an 11-month-old female patient who had post caustic ingestion multiple esophageal strictures that required eight dilatations and ended with esophagectomy and gastric tube surgery. Out of 43 (93.5%) patients with available data about the use of proton pump inhibitors (PPIs), 20 (46.5%) patients received PPIs, 18 received omeprazole, and two patients received esomeprazole (13 patients with dilatations and seven without dilatations). Seven (26.9%) patients still require ongoing dilatations (four had EA/TEF repair, one had GERD, one had epidermolysis bullosa, and one had caustic stricture). On follow-up, 12 of the 16 patients who underwent EA/TEF surgery and dilatations achieved complete response and did not need further dilatation by the age of three years (IQR = 2–5). The median follow-up period was six years (IQR = 2.25–9.0).

In terms of safety, no patient had immediate complications post endoscopic dilatation; no esophageal perforation or mortality was recorded. The overall complications in the 46 patients with esophageal strictures are shown in Table 2.

Patients with esophageal dilatations had more complications compared to those without dilatation, 25 (96.2%) versus 13 (65%) (*P* = 0.014). Recurrent hospitalization (>one time) was found only in the patients who required dilatations while prolonged hospitalization (>two weeks) was noted in both groups (13 (28.3%) out of the 46 patients, four of them underwent dilatations). The first patient was a female who had multiple levels of esophageal strictures secondary to accidental corrosive ingestion that ended with surgical resection of the esophagus. The second female patient had an accidental hydrochloric acid ingestion with esophageal strictures and gastric scaring. The third patient was a male who had trisomy 21 with a severe stricture post EA/TEF repair that required redo surgery and a total of four dilatations. The last female patient had also trisomy 21 who underwent pyloric stenosis and duodenal atresia repairs with esophageal stricture secondary to gastroesophageal reflux disease.

There were no significant differences in the number of dilatations required, the success rate and complication rate in terms of the type and location of esophageal stricture, and the dilatation technique used (Table 3). There was also no significant difference in the number of dilatations and the complication rate between the two gastroenterologists who performed the procedure. Yet, there was a significant difference in the success rate between the two gastroenterologists (*P* = 0.047). The second gastroenterologist (2011–2019) had higher satisfactory and inadequate success rates.

## 4. Discussion

In the current study, anastomotic stricture post EA/TEF repair was the main indication for endoscopic esophageal dilatations. This is similar to several studies published from neighboring countries to Bahrain and worldwide [1, 3, 8, 10, 14, 17]. The median number of esophageal dilatations in this study was three sessions per patient. This was also comparable to the worldwide published figures which are ranging between 2 and 4 sessions per patient [1, 2, 7–9, 13, 15–17, 20–22]. However, few studies reported a higher median number of dilatations reaching 5–7.5 sessions per patient [3, 10, 14] (Table 4).

SGB was the main dilator used for the procedure in the present study, in 21 (80.8%) patients. However, most of the reviewed studies were using EBD alone [3, 8, 10, 14, 16, 20, 22, 24], or in combination with SGB [1, 7, 9, 17, 23]. Few studies used SGB alone to dilate esophageal strictures [2, 13, 15]. This difference in the type of dilator used can be explained by the unavailability of smaller sizes of balloon dilators in our institution, as the minimum size was of 8 to 10 mm diameter which cannot be passed in a very narrow lumen of infants which represent most of our patients (86.5%, 32/37 patients). The choice of esophageal dilatation technique (SGB or EBDs) is influenced by the type of stricture, local expertise/preference, device and fluoroscopy availability, and the cost of each procedure [1, 23]. SGB can be reused so they are more cost effective, whereas balloon dilators are proposed for single use only [9]. Yet, SGB may apply a sudden shearing axial force and subsequently increases the risk of esophageal trauma while EBD has the benefit of applying a gradual uniform radial force on the area of the stricture [1, 22]. Balloon dilatation is commonly used in adults and its use in children increased in the last two decades [3, 14]. A study from France suggested that EBD is an effective therapy for esophageal strictures in children, with a success rate ranging between 76% and 100% based on the stricture etiology [22]. A study from Taiwan showed an improvement in the nutritional status of all the 50 studied children with esophageal stricture who underwent EBD [8]. The combined method of SGB and EBD may result in fewer dilatation repeats [17].

In some patients, treatment of benign esophageal strictures might be challenging and time-consuming [25]. Multiple dilatation sessions may be required to efficiently treat esophageal strictures and avoid recurrence [2, 6, 10, 24]. In this study, 21 (80.7%) patients required more than one dilatation. Patients with severe strictures need more frequent dilatations than those with mild strictures [23]. In the present study, patients with nonanastomotic strictures had more frequent dilatations compared to those with anastomotic strictures (*P* = 0.007). Similarly, Reinders and Wyk reported a higher average number of dilatations in patients with caustic strictures compared to those with esophageal atresia [17]. The Cakmak et al. study also showed that the corrosive strictures are longer and needed a higher number of dilatation sessions compared to anastomotic strictures [3]. The Chang et al. study revealed that all patients with esophageal strictures due to alkaline corrosive ingestion affecting long segment or multiple sites had a significantly higher rate of failure of EBD therapy [8]. In patients with caustic ingestions, especially those with significant scarring in the beginning, scarring can develop with each dilatation [24]. Alshammari et al. reported that 50% of corrosive injury cases developed restenosis. This could be due to long-lasting local inflammation and fibrous tissue formation after caustic ingestion [22]. Stricture recurrence after dilation is frequently observed in both caustic and peptic strictures [13]. GERD-related strictures can be severe and difficult to manage, particularly in patients with a prolonged history of GERD [1]. One or several dilatations are effective for most peptic strictures but it is often insufficient for caustic strictures [6].

The success rate of dilatation varies between different studies according to the underlying etiology, the method used for dilatation, and the criteria used to define effectiveness [2, 3, 14, 22–24]. The efficacy rate was better with strictures that developed post EA/TEF repair (67.4 to 100%) when compared to peptic (50 to 97%) and caustic (14 to 76.8%) strictures on reviewing the literature(see Table 4). Esophageal dilatations using balloon catheter, SGB, or a combination of both are safe, effective, and well-tolerated procedures and have high clinical success rates in children with esophageal strictures [3, 8, 9, 17, 22]. However, in this study, there were no significant differences in the success rate in terms of type and location of esophageal stricture and dilatation technique. This finding was supported by the results of the literature review as shown in Table 4. It was difficult to link the efficacy rate with the type of dilator used. The efficacy rate was ranging between 50 and 100% with SGD alone, 14 to 100% with EBD alone, and 67.4 to 98.8% if both dilators were used. Yet, this finding might be explained by the small sample size in our study. However, there was a significant difference in the success rate between the two physicians who performed the procedure (*P* = 0.047). This difference might be attributed to the variation in the personal skills between the two gastroenterologists and to the fact that some of the patients of the second gastroenterologist still need further follow-up to decide their final outcome.

In the current study, the success rate of endoscopic esophageal dilatations was 98.8%. Most of the reviewed studies showed an efficacy rate that ranges between 90 and 100%, as shown in Table 4 [1, 2, 7, 10, 14, 15, 17]. Similar to our study, the Reinders and Wyk study on 63 children showed a success rate of 98.8% (427 out of 432 dilatations performed) [17]. Very few studies reported an efficacy rate of less than 70% [13, 23]. For example, in the Scolapio et al. study on 251 adults with benign esophageal strictures, 69% of patients showed instant benefit after dilatation. The success rate was indistinguishable for both the SGB- and EBD-treated patients [23].

In this study, seven (26.9%) patients still require ongoing dilatations. Similarly, Al Sarkhy et al. reported that 13 (30.2%) out of 43 patients required ongoing dilatation [1]. Allmendinger et al. reported that two (25%) out of eight patients still need further dilatations [10]. However, most of the reviewed literature did not report the number of patients who still require dilatations at the time of their study [2, 3, 9, 11, 14–17, 22, 26].

In terms of complications post esophageal dilatation, patients are expected to have slight chest pain, nausea, vomiting, and a small amount of hematemesis that resolve within a short period of time [7]. In the present study, no patient had immediate post dilatation complications. Reinders and Wyk and Scolapio et al. studies showed no complications with either balloon or SGB dilatation methods [17, 23].

Perforation of the esophagus is a serious complication of esophageal dilatation [15]. Iatrogenic perforations are not unusual and their incidence is about 5% [22]. No patient in the current study had esophageal perforation secondary to the endoscopic dilatation. In Al Sarkhy et al. study, three (1.7%) patients had transmural esophageal leak, all after using semirigid dilators [1]. A study by Lakhdar-Idrissi et al. also reported perforation in two out of 60 children with benign strictures [13]. In the Zouari et al. study on 11 children with peptic esophageal stricture, a single perforation was reported [9]. Severe alkaline corrosive strictures tend to have a higher risk of perforation than strictures due to other causes (up to 15%) [8, 13]. The Chang et al. study reported that perforation in five (10%) children with esophageal stricture who had EBD; 80% (4/5) was due to corrosive esophagitis [8].

Patients who required dilatations in the current study had more overall number of complications compared to those without dilatations (*P* = 0.014). Recurrent hospitalization and dysphagia were frequently reported. Likewise, Weintraub et al. reported 99% recurrent dysphagia, despite of the 100% success rate [14].

## 5. Study Limitations

Like any retrospective studies, this study was limited by missing some data related to patients' demography. Other limitations are being a single center study with a small sample size. Subsequently, comparing the safety and efficacy of endoscopic dilatations in terms of the type of dilator used (SGD versus EBD) might not be appropriate. Another limitation of this study was the lack of the use of adjuvant therapies such as intralesional steroids, mitomycin-C, endoscopic incisional therapy, or esophageal stent placement which can be used in conjunction with esophageal dilatation. Despite these limitations, this study is important being the first study from Bahrain to shed the light on the treatment modalities for esophageal strictures in the pediatric age group. It is crucial to know the efficacy and success rate of endoscopic esophageal dilatation in any referral center. Despite being operator dependent, this study showed that this type of procedure is effective and safe to be performed in pediatric patients with esophageal strictures. These findings are valuable confirmation of previous studies published from tertiary care settings in other countries. In addition, the results of this study give confidence to the patients, their guardians, and the physicians who are doing the procedure. Furthermore, this study can form a foundation for any future studies.

## 6. Conclusions

This study showed that most of patients with esophageal strictures require dilatation. Endoscopic dilatation in children with esophageal strictures is effective and safe. Yet, the success rate is operator dependent. Nonanastomotic strictures required more dilatations compared to anastomotic strictures. Findings of this study are comparable to those reported worldwide. Further studies are needed to find the best treatment modality of esophageal strictures and its long-term impact on patients' quality of life.

## Figures and Tables

**Table 1:** Demographic data of 46 children with esophageal strictures with or without endoscopic esophageal dilatation.

Demographic data	Patients with esophageal strictures (*N* = 46)		*P* value^∗^ (95% CI^∗∗^)
	Dilatation *N* = 26 (56.5)	No dilatation *N* = 20 (43.5)	
Gender (*N* = 46)			
Male	15 (57.7)	10 (50)	0.604
Female	11 (42.3)	10 (50)	
Nationality (*N* = 46)			
Bahraini	21 (81)	15 (75)	0.453
Non-Bahraini	5.0 (19)	5.0 (25)	
Gestational age (*N* = 39)			
Term	14 (73.7)	15 (75)	0.925
Preterm	5.0 (26.3)	5.0 (25)	
Type of delivery (*N* = 39)			
NVD^†^	15 (78.9)	10 (50)	0.060
LSCS‡	4.0 (21.1)	10 (50)	
Birth weight (kg), mean ± SD^§^ (*N* = 38)	2.61 ± 0.68	2.68 ± 0.59	0.701 (−0.34–0.50)
Age at presentation (yr), median (IQR^¶^) (*N* = 37)	0.0 (0.0–1.34)	0.0 (0.0–0.003)	0.167
Presentation age category (yr) (*N* = 37)			
0–1	15 (75)	17 (100)	0.178
>1	5.0 (25)	0.0 (0.0)	
Age at time of study (yr), mean ± SD (*N* = 45)	9.2 ± 5.39	4.2 ± 6.53	0.318 (−6.32–2.10)
Age at time of study category (yr) (*N* = 46)			
0–4	5.0 (19.2)	11 (55)	0.144
5–9	10 (38.5)	5.0 (25)	
10–14	7.0 (26.9)	3.0 (15)	
15–18	1.0 (3.8)	0.0 (0.0)	
>18	3.0 (11.5)	1.0 (5.0)	
Causes of esophageal stricture (*N* = 46)			
EA/TEF^ǁ^	16 (61.5)	19 (95)	**0.013**
Others^††^	10 (38.5)	1.0 (5.0)	
Presence of associated diseases^‡‡^ (*N* = 20)	10 (38.5)	12 (60)	0.280

Values are presented as number (%), mean ± standard deviation, or median (interquartile range).^∗^Pearson chi-square or Fisher's exact test was used for categorical variables, while Mann–Whitney *U* or Student's *t*-test was used for continuous variables. ^∗∗^95% confidence interval. Boldface indicates a statistically significant difference with *P* < 0.05. ^†^Normal vaginal delivery; ^‡^lower segment caesarean section; ^§^standard deviation; ^¶^interquartile range; ^ǁ^esophageal atresia/tracheoesophageal fistula; ^††^seven patients with gastroesophageal reflux, two postcorrosive ingestion, one epidermolysis bullosa, and one eosinophilic esophagitis (did not require dilatation); ^‡‡^congenital anomalies involving cardiovascular, pulmonary, gastrointestinal, genitourinary, and skeletal systems, and congenital syndromes; two patients had two associated diseases each.

**Table 2:** Complications in 46 children with esophageal strictures with or without esophageal dilatations.

Variables	Patients with esophageal strictures (*N* = 46)			*P* value^∗^
	Dilatation *N* = 26 (56.5)	No dilatation *N* = 20 (43.5)	Total *N* = 46 (100)	
Overall complications	25/26 (96.2)	13/20 (65)	38/46 (82.6)	**0.014**
Recurrent hospitalization (>1 time)	22/26 (84.6)	0.0/20 (0.0)	22/46 (47.8)	**<0.0001**
Dysphagia	14/23 (60.9)	2.0/18 (11.1)	16/41 (39)	**0.001**
Gastroesophageal reflux	13/24 (54.2)	8.0/19 (42.1)	21/43 (48.8)	0.543
Failure to thrive (thinness)	4.0/19 (21.1)	7.0/16 (43.8)	11/35 (31.4)	0.273
Stunting	4.0/9.0 (44.4)	2.0/13 (15.4)	6.0/22 (27.3)	0.178
Pneumonia/aspiration	4.0/23 (17.4)	6.0/19 (31.6)	10/42 (23.8)	0.468
Hospitalization (>2 weeks)	4.0/26 (15.4)	9.0/20 (45)	13/46 (28.3)	**0.046**
Apnea/dying spells	2.0/26 (7.7)	4.0/20 (20)	6.0/46 (13)	0.380

Values presented as number (%). ^∗^Fisher's exact test was used to compare the categorical variables. Boldface indicates a statistically significant difference with *P* < 0.05.

**Table 3:** Outcome of endoscopic dilatations in children with esophageal strictures in relation to the type and location of stricture, dilatation technique, and gastroenterologist who performed the procedure.

Variables	Patients *N* = 26 (56.5)	Dilatations *N* = 88 (100)	*P* value	Success rate			*P* value	Complication rate *N* = 25 (96.2)	*P* value
				Complete *N* = 18 (69.2)	Satisfactory *N* = 7 (26.9)	Inadequate *N* = 1 (3.9)			
Type of stricture									
Anastomotic	16 (61.5)	39 (44.3)	0.517^∗^	6.0 (33.3)	3.0 (42.9)	1.0 (100)	0.395^†^	15 (60)	0.615^§^
Nonanastomotic	10 (38.5)	49 (55.7)		12 (66.7)	4.0 (57.1)	0.0 (0.0)		10 (40)	
Location of stricture									
Upper	18 (69.2)	63 (71.6)	0.749^‡^	12 (66.7)	5.0 (71.4)	1.0 (100)	0.429^†^	17 (68)	0.794^†^
Middle	7.0 (27)	23 (26.1)		6.0 (33.3)	1.0 (14.3)	0.0 (0.0)		7.0 (28)	
Lower	1.0 (3.8)	2.0 (2.3)		0.0 (0.0)	1.0 (14.3)	0.0 (0.0)		1.0 (4.0)	
Dilatation technique									
Savary dilators	21 (80.8)	76 (86.4)	0.663^‡^	15 (83.3)	5.0 (71.4)	0.0 (0.0)	0.484^†^	20 (80)	0.884^†^
Balloon dilators	3.0 (11.5)	8.0 (9.1)		1.0 (5.6)	2.0 (28.6)	1.0 (100)		3.0 (12)	
Both (Savary and balloon)	2.0 (7.7)	4.0 (4.5)		2.0 (11.1)	0.0 (0.0)	0.0 (0.0)		2.0 (8.0)	
Gastroenterologist									
1 (1995–2010)	9.0 (34.6)	29 (32.9)	0.634^∗^	9.0 (50)	0.0 (0.0)	0.0 (0.0)	**0.047** ^†^	8.0 (32)	0.346^§^
2 (2011–2019)	17 (65.4)	59 (67.1)		9.0 (50)	7.0 (100)	1.0 (100)		17 (68)	

Values presented as number (%). ^∗^Mann–Whitney *U*-test and ^‡^Kruskal Wallis test were used to compare continuous variables with categorical variables. ^†^Pearson chi-square test and ^§^Fisher's exact test were used to compare the categorical variables. Boldface indicates a statistically significant difference with *P* < 0.05.

**Table 4:** Summary of studies of patients with esophageal stricture that require dilatations from neighboring countries and worldwide.

Country	Author, year	*N*	Age (Y)	Main etiology	Esophageal dilatation					
					Dilator type	*N*	Median	Range	Efficacy (%)	Perforation
Bahrain^†^	Isa et al., 2021	26/45	<18	EA/TEF	SGB, EBD, both	88	3	1–8	98.8	0
Saudi Arabia	Al-Hussaini., 2016[15]	11/50	4–12	EoE	SGB	19	2	1–3	100	0
Saudi Arabia	Al Sarkhy et al., 2018[1]	43	2–17	EA/TEF	EBD, SGB	180	3	1–48	67.4	3
Iran	Dehghani et al., 2019[16]	82	15 d–14	Caustic	EBD	NR	2.4	1–10	76.8	4
Turkey	Cakmak et al., 2014[3]	38	0–14	EA/TEF	EBD	NR	5	1–37	86.8	4
China	Kabbaj et al., 2011[2]	72	18–75	Peptic	SGB	662	2	2–7	97	0
China	Zhang et al., 2013[7]	13	7–54	Caustic	SGB, EBD	112	3.5	1–27	92	0
Taiwan	Chang et al., 2018[8]	50/69	<18	EA/TEF	EBD	268	3	1–33	72	7
Korea	Youn et al., 2010[20]	14	17–85 m	Caustic	EBD	52	4	1–8	14–33	2
Tunisia	Zouari et al., 2014[9]	11	Median 2	Peptic	SGB, EBD	44	4	1–21	NR	1
Morocco	Lakhdar-Idrissi et al., 2012[13]	60	10 m–17	Peptic	SGB	247	4.1	1–15	50–70	2
South Africa	Reinders and Wyk, 2014[17]	63	≤12	EA/TEF	EBD, SGB, both	432	4–11	NR	98.8	0
Netherlands	Van der Zee and Bax, 2007[21]	22/51	Neonate	EA/TEF	NR	NR	1.5	1–18	NR	3
France	Alshammari et al., 2011[22]	49	<18	EA/TEF	EBD	138	2	1–8	86	3
USA	Allmendinger et al., 1996[10]	8	2 m–14	EA/TEF	EBD	62	7	1–16	100	0
USA	Scolapio et al., 1999[23]	251	Adults	Schatzki ring	EBD, SGB	277	NR	NR	69	0
USA	Yeming et al., 2002[24]	20	17 d–12	EA/TEF	EBD	126	NR	1–40	85	1
USA	Wintraub et al., 2006[14]	49	18 d–18	EA/TEF	EBD	272	5.6	1–32	100	1

^†^The current study; EA/TEF: esophageal atresia/tracheoesophageal fistula; SBG: Savary-Gilliard bougienages; EBD: endoscopic balloon dilator; EoE: eosinophilic esophagitis; NR: no record; USA: United States of America.

## Data Availability

Data will be available upon request anytime.
